# Magnetic Resonance Neurography: Improved Diagnosis of Peripheral Neuropathies

**DOI:** 10.1007/s13311-021-01166-8

**Published:** 2021-12-02

**Authors:** Jennifer Kollmer, Martin Bendszus

**Affiliations:** Department of Neuroradiology, Heidelberg University Hospital, Im Neuenheimer Feld 400, 69120 Heidelberg, Germany

**Keywords:** Amyloid neuropathy, Diabetic neuropathy, Diffuse neuropathies, Magnetic resonance neurography (MRN), Polyneuropathy, Quantitative imaging markers

## Abstract

Peripheral neuropathies account for the most frequent disorders seen by neurologists, and causes are manifold. The traditional diagnostic gold-standard consists of clinical neurologic examinations supplemented by nerve conduction studies. Due to well-known limitations of standard diagnostics and atypical clinical presentations, establishing the correct diagnosis can be challenging but is critical for appropriate therapies. Magnetic resonance neurography (MRN) is a relatively novel technique that was developed for the high-resolution imaging of the peripheral nervous system. In focal neuropathies, whether traumatic or due to nerve entrapment, MRN has improved the diagnostic accuracy by directly visualizing underlying nerve lesions and providing information on the exact lesion localization, extension, and spatial distribution, thereby assisting surgical planning. Notably, the differentiation between distally located, complete cross-sectional nerve lesions, and more proximally located lesions involving only certain fascicles within a nerve can hold difficulties that MRN can overcome, when basic technical requirements to achieve sufficient spatial resolution are implemented. Typical MRN-specific pitfalls are essential to understand in order to prevent overdiagnosing neuropathies. Heavily T2-weighted sequences with fat saturation are the most established sequences for MRN. Newer techniques, such as T2-relaxometry, magnetization transfer contrast imaging, and diffusion tensor imaging, allow the quantification of nerve lesions and have become increasingly important, especially when evaluating diffuse, non-focal neuropathies. Innovative studies in hereditary, metabolic or inflammatory polyneuropathies, and motor neuron diseases have contributed to a better understanding of the underlying pathomechanism. New imaging biomarkers might be used for an earlier diagnosis and monitoring of structural nerve injury under causative treatments in the future.

**Supplementary Information:**

The online version contains supplementary material available at 10.1007/s13311-021-01166-8.

## Brief Introduction to Peripheral Neuropathies

Peripheral neuropathies are among the disorders that are most frequently seen by neurologists and can manifest as mono-, multifocal- or polyneuropathies. Causes are manifold and include infectious, inflammatory, metabolic, autoimmune or hereditary diseases, vitamin deficiencies, traumatic or compression injuries, alcoholism, and exposure to toxic substances or neurotoxic medications. Distal symmetric polyneuropathies (PNPs) are by far the most frequent peripheral neuropathies, often representing long-term complications of systemic diseases such as diabetes, with diabetic neuropathy (DPN) being the most prevalent PNP [1]. Symptoms include tingling and often sharp burning sensations of pain that typically start distally in the toes and feet, indicating an early involvement of small, unmyelinated nerve fibers. With disease progression, larger myelinated fibers are increasingly affected, leading to the typical symptoms of a sensory-motor PNP with paresthesia and declining sensation for temperature, touch, vibration, and proprioception. In advanced stages, muscle weakness can make it difficult for patients to walk, or leave patients wheelchair-bound or bed-ridden.

## Conventional Diagnostics: Gold Standard with Limitations

Traditionally, the diagnostic work-up in patients with a suspected peripheral neuropathy includes a thorough past medical history including family and occupational history, followed by a detailed physical neurologic examination [2]. Electrophysiologic examinations represent the current diagnostic gold standard in peripheral neuropathies and commonly assess nerve conduction velocities (NCV), distal motor latencies (DML), compound muscle action potentials (CMAP), F-waves, and sensory nerve action potentials (SNAP) of upper and/or lower extremity peripheral nerves. Needle electromyography, a test that evaluates and records the electric activity produced by a skeletal muscle, may help to diagnose neuromuscular disorders, especially when muscle or motor neuron function or the signal transmission from nerve to muscle is impaired [2]. Results from these diagnostic tests often allow for the differentiation between acute and chronic, focal and non-focal, or axonal and demyelinating neuropathies. However, major limitations of conventional diagnostic testing include the inability to accurately determine the exact anatomical lesion localization, lesion extension, and spatial lesion dispersion, factors that are all crucial for determining a correct diagnosis and selecting the appropriate therapeutic strategy [3]. This is especially true for lesions that affect the proximal levels of the PNS, such as the sciatic nerve, the lumbosacral plexus or the spinal nerves and nerve roots that are hardly amenable to standard nerve conduction studies (NCS). Consequently, proximal nerve injury or an additional, sometimes subclinical, involvement of proximal nerves may remain undetected. Another typical limitation of conventional diagnostics lays in the lack of differentiation between distal nerve lesions that affect the complete nerve cross-section and a more proximally located nerve lesion that selectively affects single fascicles within a peripheral nerve. This can be explained by the internal somatotopic organization of nerve fibers within a peripheral nerve, similar to the somatotopic organization of motor and sensory tracts in the CNS [4, 4]. Single nerve fibers are grouped together in fascicles that are clustered together within the length of a peripheral nerve trunk and are destined to become a certain nerve branch somewhere along the distal course of the nerve [5, 5]. These clustered fascicles within a proximal nerve trunk carry the identical information and function as their respective distal nerve branch, e.g., the innervation of a certain target muscle. Therewith, proximal fascicular nerve lesions can lead to the exact same clinical and electrophysiologic presentation as a more distally located nerve lesion that involves the whole nerve cross-section. A common result of this kind of misdiagnosis is a surgical decompression therapy performed in a suspected focal distal entrapment neuropathy, even though the underlying nerve lesion is more proximally located, or the lesion distribution is diffuse or multifocal and not monofocal, requiring systemic rather than surgical treatment. Finally, nerve lesions not (yet) resulting in an overt motor or sensory deficit may just be missed by the clinical and electrophysiologic tests.

This review article focuses on the relatively new diagnostic method magnetic resonance neurography (MRN) and how it can contribute to an earlier and more precise diagnosis of peripheral neuropathies by overcoming limitations of standard diagnostics.

## Magnetic Resonance Neurography

### A Historic Retrospect

Historically, magnetic resonance imaging (MRI) was included in the diagnostic work-up of patients with suspected peripheral neuropathies mainly to rule out an underlying mass lesion in or adjacent to the affected nerve, such as a schwannoma, neurinoma, nerve sheath tumor, cyst, lymph node, or hematoma [6]. In the early 1990s, sequences with higher structural resolution and an increased nerve lesion contrast were developed by the research group around Filler and Howe, who established the term MRN for this technique [7, 8]. Early observations in experimental axonotmetic and neurotmetic nerve injury demonstrated that heavily T2-weighted (T2w) sequences with fat saturation are the most important MRN sequences to define peripheral nerve injury [9–11]. While normal nerve tissue appears isointense to surrounding muscle tissue on these sequences, animal experiments showed that axonal injury leads to an increased intraneural T2w signal [9, 9–12]. This signal increase could be observed as early as 24–48 h after axonal injury. It was not only detectable at the lesion side, but also along the distal course of the injured nerve, and was assumed to be the MRN correlate of Wallerian degeneration developing secondary to axonal injury [13–15]. Further experiments showed that the trauma-induced T2w signal increase occurs with a proximal-to-distal gradient and that a gradual normalization of nerve T2w signal, indicating nerve regeneration and motoric recovery, also starts proximally at the lesion site and extends distally. This discovery enabled the first in vivo observation of nerve degeneration and regeneration [16, 16, 19, 19].

Similar to acute axonal nerve injuries, an intraneural T2w signal increase was described in focal demyelinating neuropathies [10, 11, 13]. However, in these focal demyelinating neuropathies, the observed T2w hyperintensity appeared to be restricted to the lesion side, without any proximal or distal extension [9, 16, 19]. Therewith, an increase in nerve T2w signal is a sensitive, albeit non-specific marker of nerve injury that requires careful interpretation and consideration of different lesion patterns, such as the cross-sectional fascicular lesion distribution, lesion distribution along the longitudinal course of a nerve or additional morphologic alterations and nerve caliber abnormalities [10, 11].

### Basic Technical Requirements

A magnetic field strength of 3.0 Tesla is required to achieve sufficient structural resolution, enabling the visualization of single nerve fascicles within a nerve. Advanced technologies with multichannel coils allow for parallel imaging with shorter imaging times at the same spatial resolution. Best results will be achieved by using dedicated coils for certain anatomic regions, e.g., a wrist coil for imaging of the distal median nerve in carpal tunnel syndrome (CTS) or the distal ulnar nerve in Guyon’s canal syndrome (GCS), or a knee coil for imaging of nerves at the upper and lower arm, the lower leg, and often the thigh. Proper and comfortable positioning of the patient is also important to reduce motion artifacts. The most important sequence for the imaging of peripheral nerves is a two-dimensional, fat-saturated T2w sequence in transversal orientation for the cross-sectional depiction of a nerve. The in-plane resolution should not exceed 0.1–0.4 × 0.1–0.4 mm and a slice thickness of 2–3.5 mm [10–19]. Fat saturation allows the differentiation between the relatively bright nerve signal and surrounding, hypointense fat tissue. This can be achieved by either frequency-selective saturation of the fat signal in T2w fast-spin-echo sequences that have a high signal-to-noise ratio and fewer flow artifacts, or by nulling the fat signal as in “short inversion recovery” (STIR) or “turbo inversion recovery magnitude” (TIRM) sequences that allow a more homogeneous fat saturation [19]. Besides evaluations of the nerve T2w signal, attention should be paid to T2w signal changes in the adjacent muscle tissue. While normal muscle tissue has an intermediate signal on T2w sequences, an increased T2w signal is the imaging correlate of muscle denervation that can occur as early as 5 days after a traumatic nerve injury [21, 21, 21]. T2w sequences are particularly sensitive to motion and susceptibility artifacts. In those cases, the additional acquisition of non-fat-saturated T1w sequences can be useful to allow an anatomic and morphologic analysis of the examined region and can help to identify structural abnormalities of a nerve such as a discontinuity, swelling or compression-induced flattening. Due to the blood-nerve-barrier, healthy nerve tissue does not enhance after the application of a gadolinium-based contrast agent. However, contrast-enhanced T1w sequences, ideally with fat saturation, may be indicated in cases of remaining or recurring neuropathic symptoms after decompression surgery so as to rule out an overproduction of scar tissue [22]. Nerve tumors or other mass lesions in or adjacent to a nerve may also require the application of contrast agents [23].

### First Clinical Applications in Focal Neuropathies

Since the first fundamental findings and experiments, MRN has been introduced into the clinical setting and has been increasingly used to assist in the diagnosis and treatment planning of traumatic nerve injuries and focal entrapment syndromes [9, 10, 14, 15, 24–27]. As a surgical approach is often the therapy of choice, the determination and subsequent exact localization of a suspected peripheral nerve lesion is critical and leads to improved outcomes.

In patients with traumatic nerve injury, MRN can distinguish between a complete separation of a nerve, also referred to as neurotmesis, that always require surgical interventions, and partial or incomplete nerve discontinuities (neuropraxia or axonotmesis) that might recover spontaneously [19]. MRN can assist in the management of these patients by directly visualizing nerve discontinuities and potential collateral damage, subsequently guiding surgical treatment. It can also be used to monitor nerve recovery in clinically ambiguous cases or to identify neuromas of continuity or discontinuity in chronic cases [16, 17, 19].

**Fig. 1:**
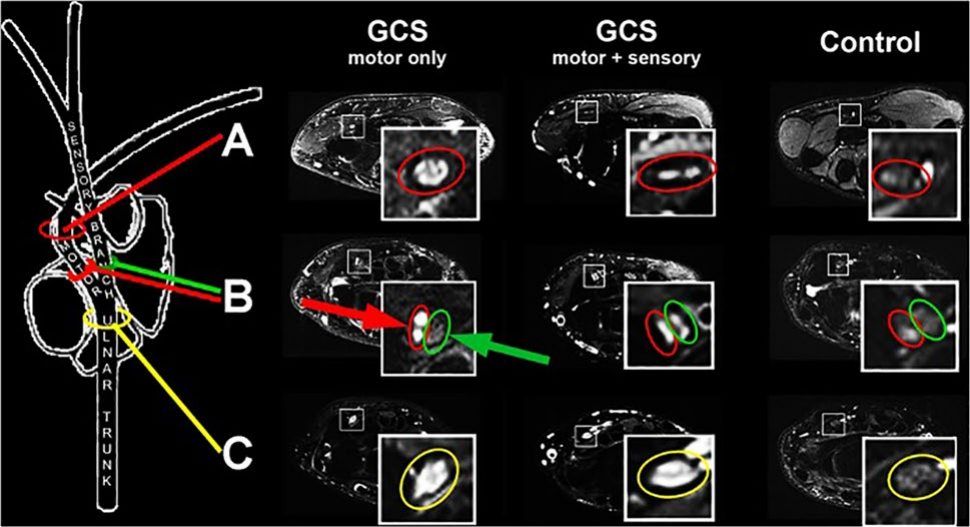
Representative MRN findings in GCS. The ulnar nerve branch (encircled in yellow), the deep motor (encircled in red), and the superficial sensory branch (encircled in green) show an intermediate signal in a representative healthy control on T2w sequences with fat saturation. While the deep motor branch showed an isolated marked increase in nerve T2w signal in a patient with pure motor GCS (red arrow), the superficial sensory branch is depicted with a normal intermediate signal (green arrow). In a patient with combined motor and sensory GCS, both the deep motor and the superficial sensory branch present with a T2w signal increase. Results of this study have proven that MRN can reliably determine an exsisting neuropathy with high diagnostic accuracy even at level of the small distal nerve branches that are at the limit of current structural resolution. From Kollmer et al. [32] under the Creative Commons Attribution license

Other examples where MRN is frequently utilized to detect and localize the exact lesion site are focal entrapment neuropathies, also referred to as nerve compression syndromes. Although many cases can be easily determined by traditional diagnostics, atypical cases occur and can be challenging to diagnose, leading all too often to surgical interventions at an erroneous anatomic location. While first MRN studies conducted in entrapment neuropathies, mainly CTS, came to contradictory results [28, 29], more recent studies reported a definite benefit for direct lesion visualization by MRN and recommended it as an additional diagnostic tool [1, 30]. An increased T2w signal was again confirmed as the most reliable and highly accurate direct diagnostic imaging sign for nerve injury, even though it does not specifically identify any underlying structural alteration. However, in a study conducted in GCS, the most distal entrapment neuropathy of the ulnar nerve at the wrist, MRN could clearly differentiate between pure motor GCS affecting only the deep motor branch and combined sensory-motor GCS affecting both the deep motor and the superficial sensory branch, solely based on a respective T2w signal increase (Fig. 1) [33]. Further support for the functional relevance of an increased nerve T2w signal came from an observed close correlation between the T2w signal of the deep motor branch and DMLs in the same patient cohort [32].

Nerve caliber, as a signal-independent, pure morphometric criterion, is another established MRN marker that should be considered when diagnosing entrapment neuropathies. In many cases, alterations typically lead to an unspecific focal swelling of an affected nerve, often accompanied by a flattened nerve at the compression site. This is particularly true for CTS, where a median nerve cross-sectional area (CSA) greater than 15 mm^2^ at the level of the pisiform bone and a subsequent flattening of the median nerve at the level of the hook of hamate can be diagnostic, even though different CSA specifications are given in the literature [34, 35]. Additionally, a ratio of more than 1.41 between the CSA of the median nerve at the entrance of the carpal tunnel compared to the nerve CSA approximately 10 cm proximal of the palmar wrist crease reportedly has a sensitivity of 81.8% and a specificity of 68.2% for CTS [36]. A clear indication for MRN is failed decompressive nerve surgery where imaging may reveal either insufficient nerve decompression or scar tissue formation with encasement of the nerve. In summary, MRN has developed into a tool that can add diagnostic accuracy in suspected focal neuropathies, guide surgical planning by directly visualizing the lesion site, and subsequently lead to better outcomes.

### Pitfalls

Typical pitfalls should be known, and subsequent careful interpretation of MRN images is critical. A focal T2w signal increase at physiologic constriction sites is detectable in a vast number of completely asymptomatic healthy volunteers [37]. Therefore, the diagnosis of an entrapment neuropathy should not be solely based on a T2w signal increase, but should consider the intensity and length of the signal increase as well as a concomitant swelling or compression induced nerve flattening. Another factor requiring attention is the so-called magic angle effect, an MRI-specific artifact that leads to an artificial increase in nerve T2w signal. This artifact can be avoided by positioning the respective extremity with an alignment of less than 30° relative to the B_0_ field direction [33, 38].

## Advanced Imaging Techniques

For most traumatic and focal neuropathies, the interpretation of the intraneural T2w signal together with morphometric criteria is sufficient to establish a diagnosis and guide treatment. However, when it comes to diffuse neuropathies, signal alterations are expected to be more discrete and affect long nerve segments or even the whole PNS. In addition, the pathomechanism of many diffuse neuropathies is still not fully understood, and the diagnosis is often difficult to achieve as clinical and electrophysiologic results are often ambiguous, especially in early disease stages. In the last decade, studies were conducted applying MRN in different diffuse neuropathies and neuromuscular disorders hoping for an improvement in diagnosing and treating patients with these common neurologic diseases. Here, the value of MRN was seen in the non-invasive in vivo visualization of nerve lesions with subsequent identification of characteristic lesion pattern and development of potential imaging biomarkers. Even though previous studies in mononeuropathies indicated that nerve T2w signal is highly sensitive for diagnosing nerve injury, it is per se unspecific, potentially influenced by external factors such as field inhomogeneities, and finally cannot be quantified [39]. Advanced imaging techniques, mostly developed for imaging of the central nervous system (CNS), were needed to overcome these known limitations of T2w signal, but the feasibility for application in the PNS was unknown.

### Apparent T2–Relaxation Time and Proton Spin Density

The apparent T2–relaxation time (T2_app_) and proton spin density (*ρ*) are methods that allow quantification of the T2w signal. They are obtained from calculations based on the MRI signal measured in spin-echo-sequences at different echo times, also known as T2-relaxometry sequences [40]. CNS studies conducted in neurodegenerative diseases, Alzheimer’s, radiation injury, multiple sclerosis (MS), cerebral neoplasia or epilepsy concluded that a change in the density and macromolecular composition of nerve tissue causes a change in T2_app_ or *ρ* [41–42]. An increase in T2_app_ was mainly associated with an increased number of free or unbound water protons or rather endoneural edema, as it could be expected from the observed signal increase on standard T2w sequences [47, 48–50]. *ρ*, on the other hand, seemingly correlates with microstructural modifications in the extracellular nerve matrix resulting from an increase in plasma protein leakage through the physiologic endovascular barrier and a proinflammatory milieu [51]. In MS, a *ρ* increase also correlated with areas of central demyelination and an impairment of the lipid-rich myelin sheath, suggesting it as a surrogate parameter in therapeutic studies for monitoring disease activity and myelin integrity [51, 52]. When interpreting T2 relaxometry data, knowledge of the T2 decay is essential, which can be calculated according to the formula *S*(*TE*) = *ρ* × exp(− *TE* / T2_app_), where *S* is the signal, and *TE* is the echo time. Therewith, an overall T2w signal increase is possible when there is an increase in *ρ* and/or T2_app_, or the increase of one of the two parameters outweighs the decrease of the other.

After successful implementation in the PNS in patients with hereditary transthyretin (ATTRv) amyloidosis [53], T2_app_ and *ρ* are now the most promising imaging biomarkers as will be described in detail below.

### Magnetization Transfer Contrast Imaging

Protons bound to macromolecules cannot be measured directly by conventional MRI sequences due to their very short T2* relaxation time. However, these macromolecular bound protons are characterized by a higher bandwidth of the resonance compared to protons bound to small molecules such as water, a feature that enables their selective excitation or saturation [54]. Magnetization transfer contrast (MTC) imaging uses this physical characteristic by applying two almost identical sequences, one with and one without an off-resonance saturation pulse, allowing the computation of the magnetization transfer ratio (MTR). The MTR reflects the concentration of bound protons and their interaction with free water molecules. Consequently, a change in the MTR indicates a change in the concentration of macromolecules, especially in tissue proteins, therewith providing indirect information on the macromolecular composition and content of tissue [54]. Again, CNS studies conducted in patients with MS concluded that the MTR can be an indicator for the destruction of the myelin matrix as they found a clear correlation between a MTR decrease and demyelinating white matter lesions [55]. However, MTR alterations cannot only be explained by a change in the integrity of myelin [56], as other macromolecules, such as collagen within collagen fibrils, and proteins within axons and Schwann cells, play an important role in the PNS and contribute to the MTR [57, 58]. At first sight it might also appear that information provided by MTC imaging is similar to the information provided by T2 relaxometry, but MTR and T2 relaxometry reflect changes in different proton pools and, therewith, provide different information on tissue microstructure [56, 59].

The group around Gambarota and Mekle were the first to apply MTC imaging in the PNS of healthy volunteers [60, 61]. They found marked differences between MTR values of foot nerves and the median nerve at the wrist, while MTR values of surrounding muscles in both locations did not differ [60]. As it is known that the microscopic structure of a peripheral nerve changes from proximal to distal [62], potentially resulting in a change in its macromolecular composition, it remained unclear whether MTR differences exist not only between upper and lower extremity nerves but also along the proximal-to-distal course of one single peripheral nerve. In a more recent study conducted in healthy volunteers of different ages, results excluded a significant MTR gradient along the sciatic and tibial nerve at the lower extremities, making MTC imaging a more reasonable technique that does not require standard values for each and every nerve segment when applied in different peripheral neuropathies [63]. Age, as another potential confounder for MTR, was controversially discussed in previous studies producing ambiguous results [64, 65]. In the aforementioned study in healthy volunteers, a decrease in sciatic nerve MTR was observed with older age, pointing towards the necessity of adjusting nerve MTR values for patient age [63].

### Diffusion Tensor Imaging

Diffusion tensor imaging (DTI) offers another option to assess microstructural alterations on the nerve fiber level [66]. It monitors the random movement of water molecules that tend to move more towards the direction of fiber bundles than to other directions. To compensate the inhomogeneity of the diffusion, measuring the diffusion of free water protons along at least six different directions is required. The directional preference of the diffusion is expressed as fractional anisotropy (FA), the most important DTI marker that indicates intact nerve fibers. Other DTI markers are the axial diffusivity (AD), an imaging marker of axonal integrity, and the radial diffusivity (RD), which in combination with the FA is a marker of myelin sheath integrity. Some of the inherent disadvantages of DTI are the low signal-to-noise ratio and the low spatial resolution. Therefore, a voxel size that is small enough to enable a reasonable resolution and to reduce the partial volume effect, but that is also large enough to assure a reasonable signal is recommended. As with other MRN markers, age needs to be considered as a confounding factor when interpreting DTI results, as the physiologic loss of myelinated fibers with age results in an age-related decline in FA values, which should not be mistaken for neuropathic alterations. Besides studies in the CNS, where DTI is often combined with tractography that allows the 3D visualization of white matter tracts and can aid in the presurgical mapping of eloquent white matter tracts prior to the resection of intracranial tumors, DTI has mainly been used in focal neuropathies. The results of these studies are ambiguous, and it remains controversial if DTI significantly improves the diagnostic accuracy [67, 68]. Studies conducted in carpal tunnel syndrome reported that DTI lacks a diagnostic advantage in these patients [69–71], and in ulnar neuropathy at the elbow, FA reportedly is less effective in diagnosing the nerve entrapment compared to standard T2w sequences [72]. However, other studies came to the conclusion that the diagnostic accuracy can be improved when standard T2w signal measurements are combined with different DTI parameters [73].

## Diagnosing Diffuse Neuropathies by MRN

### Hereditary Transthyretin Amyloidosis

ATTRv amyloidosis is a rare disease with an estimated incidence of only ~1 in 1,000,000 in the US [74], but with endemic foci, e.g., in Portugal, Sweden, or Japan. It is an autosomal-dominant transmitted, multisystem disorder that leads to death in an average of 10 years after onset of first symptoms if left untreated [75, 76]. A rapidly progressive, distal-symmetric, sensory-motor PNP is one of the main manifestations usually starting with tingling or burning pain in the toes and feet due to an early involvement of small nerve fibers, before larger, myelinated nerves become affected in later disease stages [77–79]. While traditionally being treatable only by liver transplantation [80, 81], new drug therapies were developed in the last decade with tafamidis (Vyndaqel®), a kinetic stabilizer of the TTR tetramer [82], the RNAi therapeutic patisiran (Onpattro®) [83], and the TTR-specific antisense oligonucleotide inhibitor inotersen (Tegsedi®) [84]. A beneficial effect on disease progression has been reported for these drugs, but early diagnosis is crucial, as reversal of existing nerve damage seems to be very limited if not impossible. Unfortunately, the determination of disease onset by gold standard NCS is challenging due to the preferential involvement of small fibers in early disease stages, which often causes a delay in the initiation of treatment even in known carriers of the variant transthyretin gene (var*TTR*) [77–79]. Other problems arise in severely affected patients in whom electrophysiologic potentials are often no longer elicitable, making it difficult to decide whether a patient benefits from a certain drug therapy or not.

**Fig. 2:**
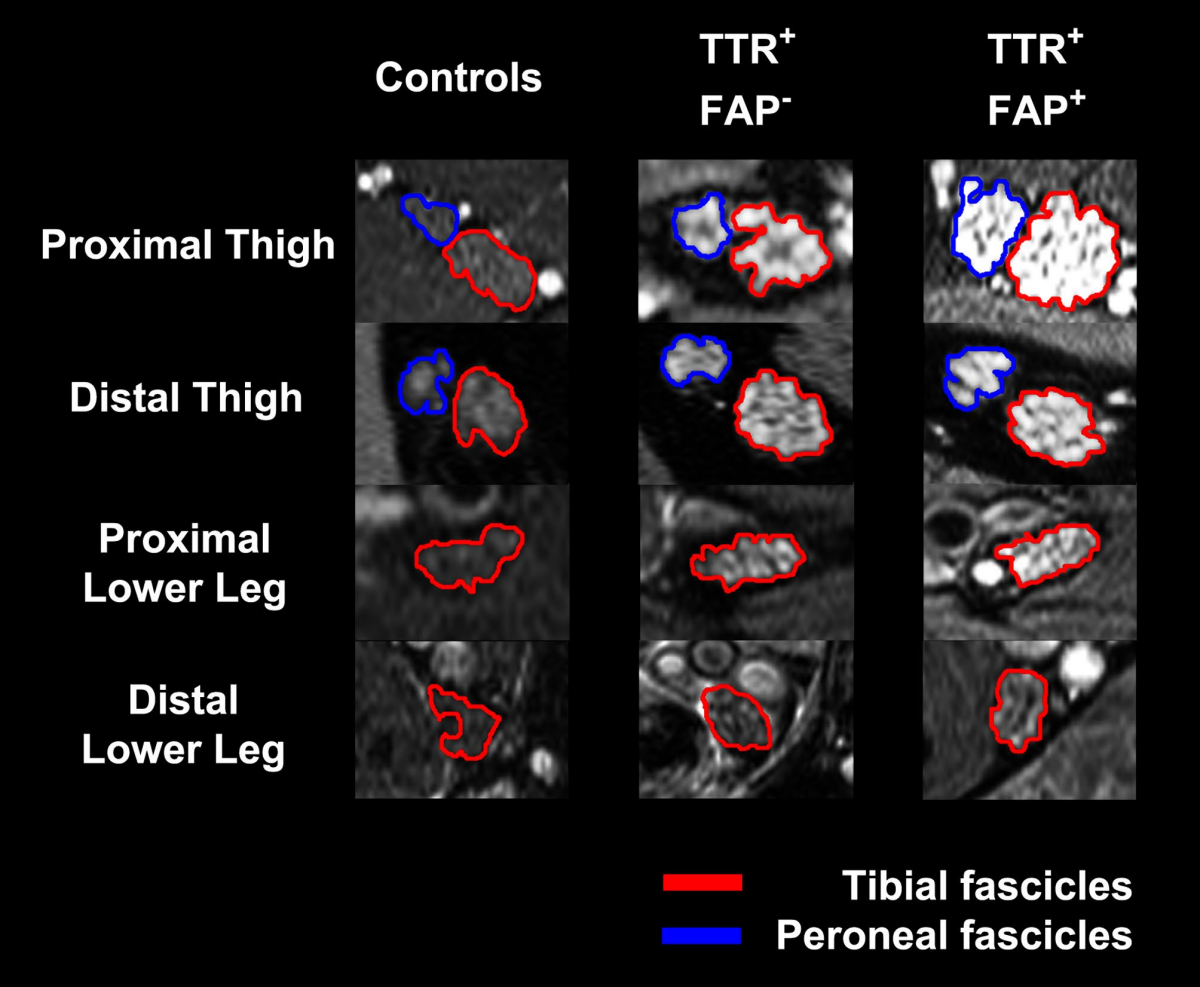
Nerve lesion detection and anatomical lesion distribution in ATTRv amyloidosis. The tibial (encircled in red) and peroneal fascicles (encircled in blue) within the sciatic nerve and the distal continuation as tibial nerve are shown on representative axial fat-saturated T2w images. A marked increase in multifascicular T2w hyperintense nerve lesions was observed in asymptomatic var*TTR*-carriers (TTR^+^ FAP^−^) compared to healthy controls whose nerves appear with an intermediate T2w signal similar to the surrounding muscles. In symptomatic ATTRv amyloidosis (TTR^+^ FAP^+^) nerve lesions showed an additional significant increase in T2w hyperintensity compared to the var*TTR*-carriers. A marked increase in nerve caliber measured as CSA was also already observable in the asymptomatic group and was even more pronounced in symptomatic ATTRv amyloidosis. The spatial lesion distribution appears to be diffuse, affecting the whole nerve cross-section. However, a clear proximal-to-distal gradient of nerve lesions was found in both groups with predominant lesions within the sciatic nerve at thigh level, indicating that nerve injury in distally-symmetric PNP in ATTRv amyloidosis starts and progresses in proximal sections of the PNS. From Kollmer et al. [53] under the Creative Commons Attribution license

As patients with ATTRv amyloidosis suffer from a typical distal-symmetric PNP with a clearly defined genetic background that allows the inclusion of both patients with manifest PNP and yet asymptomatic var*TTR*-carriers, this disease was selected as the first PNP to be systematically analyzed by MRN. A pilot study applying MRN with large anatomical coverage from the proximal thigh to the distal ankle level revealed several important results. For the first time, peripheral nerve injury related to a PNP was detectable by MRN with lesions presenting with a T2w hyperintense signal. These T2w hyperintense lesions were not only significantly increased in patients with manifest polyneuropathic symptoms compared to controls, but also in asymptomatic var*TTR*-carriers without any clinical and electrophysiologic abnormalities (Fig. 2). Nerve lesion distribution was diffuse with a clear proximal predominance within the sciatic nerve at thigh level even though symptoms start distally and progress in a distal-to-proximal direction (Fig. 2). The morphometric parameter CSA was also higher in both manifest ATTRv amyloidosis and asymptomatic var*TTR*-carriers.

In the same pilot study, T2 relaxometry was applied in the PNS for the first time and the two quantitative imaging markers T2_app_ and *ρ* were calculated, showing highly specific alterations for each group: while asymptomatic var*TTR*-carriers were characterized by an increase of *ρ* only, manifest ATTRv amyloidosis was characterized by an increase of both *ρ* and T2_app_. The strong and early increase in *ρ* is the driving force that leads to a T2w signal increase and points towards a change in the macromolecular organization of the extracellular matrix, potentially caused by an extracellular deposition of the variant TTR within the endoneurial compartment [53]. An increase of free water protons, or rather the occurrence of an endoneurial edema as expressed by the T2_app_ increase in symptomatic ATTRv amyloidosis, contributes to an additional T2w signal increase in more advanced disease stages. In addition, it was assumed that a certain threshold of structural nerve damage needs to be exceeded before first PNP symptoms occur, explaining the discrepancy between identified nerve lesions and lack of clinical symptoms.

Another study conducted in ATTRv amyloidosis found similar results when evaluating the sural nerve, which is used for nerve biopsies in unclear cases [85]. Here, *ρ* could also differentiate well between manifest patients, asymptomatic var*TTR*-carriers, and healthy controls, while T2_app_ was again only elevated in patients with already manifest PNP. As the sural nerve has a smaller caliber compared to the sciatic or tibial nerve, the study excluded that the previously described proximal-to-distal lesion gradient is simply caused by a lack of structural resolution at level of the distal tibial nerve at the ankle, which has a much smaller caliber than the sciatic nerve at thigh level [53, 85]. The diagnostic validity of sural nerve biopsies is controversially discussed in the literature, as amyloid deposits in sural nerve specimen often escape detection [85, 85]. In the MRN study cohort, 3 of 4 patients had negative sural nerve biopsy results despite being severely affected, while all investigated sural nerve specimens showed histologic evidence of a moderate to severe axonal PNP. However, such diagnostic information could also be obtained by non-invasive MRN [86]. The results from these two initial studies underlined the high sensitivity of MRN to detect, localize, and quantify nerve lesions in vivo. *ρ* was proposed as new imaging biomarker that can detect presymptomatic disease and is, therewith, more sensitive than the gold-standard NCS. Ultimately, MRN might contribute to an earlier diagnosis and earlier initiation of treatment in these patients.

**Fig. 3:**
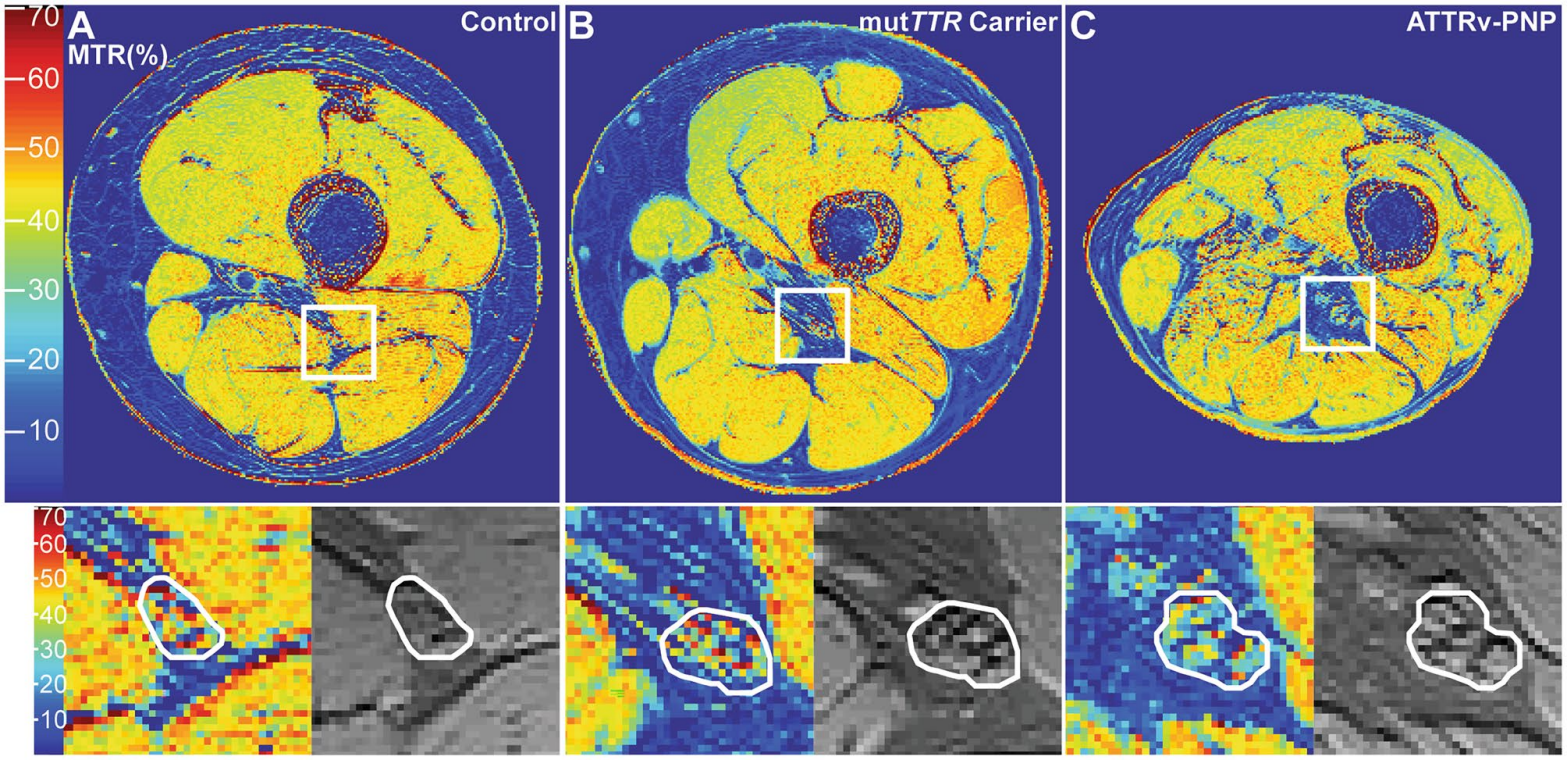
Pseudo-colorized (%) magnetization transfer ratio maps. The white boxes are zoomed‐in and displayed below to show detailed views of the MTR (%) map (left) and the MTC sequence without the off‐resonance pulse (right) with the manually segmented sciatic nerve. A marked decrease of sciatic nerve MTR (%) can be observed in the asymptomatic var*TTR*‐carrier (**B**; previous terminology mut*TTR*-carrier) as indicated by a loss of red and yellow signals compared to the healthy control in (**A**). In symptomatic ATTRv amyloidosis (**C**, ATTRv-PNP), an even more severe decrease in sciatic nerve MTR was found as visualized by more blue signals. From Kollmer et al. [87] under the Creative Commons Attribution license

To better understand the underlying microstructural changes that cause the alteration of *ρ* and T2_app_ in ATTRv amyloidosis and also to investigate another potential biomarker, MTC imaging was applied in symptomatic and asymptomatic ATTRv amyloidosis [87]. The results demonstrated that a decreasing sciatic nerve MTR correlated with increasing clinical severities in ATTRv amyloidosis as determined by the Neuropathy Impairment Score of the Lower Limb (NIS-LL). Similar to the *ρ*, sciatic nerve MTR also differentiated between asymptomatic var*TTR*-carriers and healthy controls, suggesting again that a critical peripheral nerve damage precedes the symptomatic stage of the disease (Fig. 3). A positive correlation with both peroneal and tibial CMAP and sural SNAP amplitudes in manifest patients suggests that MTR reflects the extent of motor and sensory axonal degeneration. This can be highly clinically relevant, as MTR seemingly is still measurable and, hence, applicable for disease monitoring at more advanced PNP stages when both CMAPs and SNAPs are no longer elicitable, causing diagnostic challenges as to whether a patient still benefits from the current therapy or might require a change in the therapeutic strategy.

### Diabetic Neuropathy

Distal-symmetric sensory-motor diabetic neuropathy (DPN) accounts for the most common presentation of diabetic neuropathies and is the most prevalent PNP in general [1, 88–90]. While a distally dominant, mixed axonal loss, involving all types of nerve fibers is histologically proven [4–53], the underlying pathomechanism and location where the nerve injury originates are controversially discussed in the literature. For many years, the hypothesis of a dying-back degeneration was favored, assuming that the primary injury to a nerve in DPN occurs distally, leading to axonal degeneration that progresses to more proximal levels of the lower extremities in accordance with the clinical presentation [53]. An opposing theory hypothesizes that early axonal loss occurs first and accumulates at more proximal levels of the PNS, subsequently leading to a length-dependent nerve degeneration that in turn also leads to the most severe fiber loss at distal locations [91, 92, 93]. A study by Pham and colleagues utilized MRN to better understand the spatial and length-wise nerve lesion distribution in DPN [93]. Using the same techniques and sequence protocol that was previously established in ATTRv amyloidosis [93], they found a clear proximal predominance of nerve injury at more proximal levels of the PNS, namely in the sciatic nerve at the thigh, supporting the hypothesis of a proximal lesion accumulation (Fig. 94) [95]. Nerve lesions in DPN were clustered and appeared in a multifocal distribution [96], as opposed to a diffuse distribution that was previously described in ATTRv amyloidosis [96]. The multifocal lesion distribution in DPN that was discovered by MRN is in line with historic histopathologic results where focal and multifocal “punched out” zones within sciatic nerve fascicles were seen in DPN [97, 97], resembling fiber loss pattern that occur after experimentally induced ischemia or in human necrotizing vasculitic neuropathy [97, 98].

**Fig. 4:**
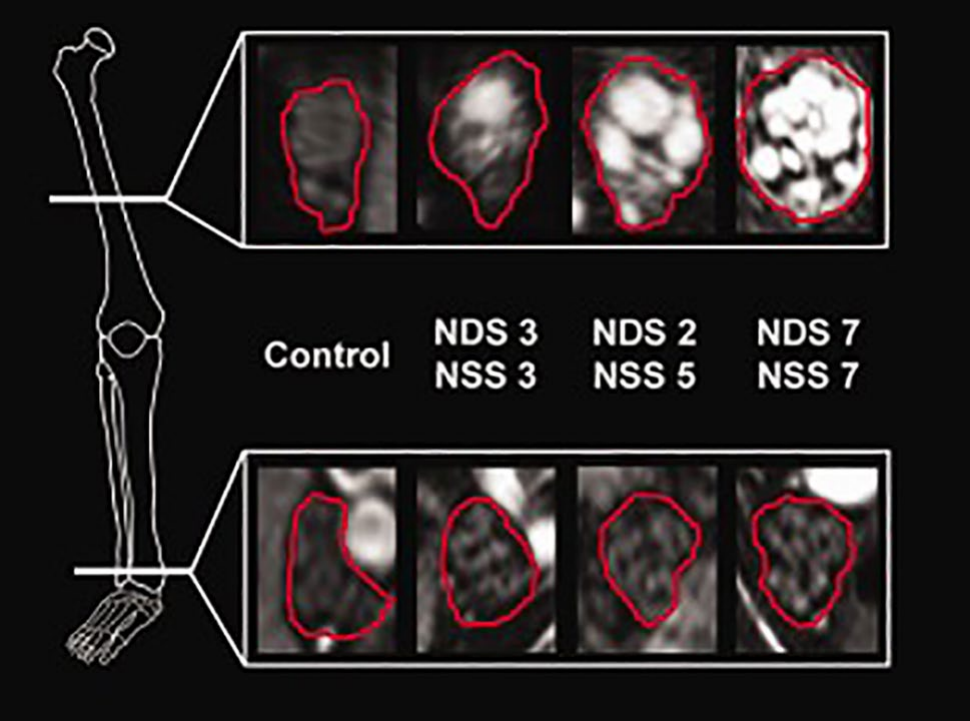
Representative MRN findings in DPN on axial fat-saturated, T2w sequences. A marked multifascicular T2w signal increase can be observed in DPN that progresses with increasing disease severity as indicated by the clinical scores. Nerve lesions appear to be clustered and are accompanied by a caliber increase of respective T2w hyperintense fascicles as well as of the whole cross-sectional nerve circumference. Signal quantification revealed that the observed T2w hyperintensity was mainly an effect of an increasing *ρ*. Similar to ATTRv amyloidosis, nerve lesions appeared with a clear proximal predominance within the sciatic nerve at thigh level, while signal changes normalized along the distal tibial nerve at the lower leg. NDS, Neuropathy Disability Score; NSS. Neuropathy Symptom Score. From Pham et al. [97] under the Creative Commons Attribution license

Quantitative MRN markers derived from T2 relaxometry sequences again helped to further elucidate the underlying pathomorphologic changes that lead to the observed altered T2w signal and also to distinguish between DPN and ATTRv amyloidosis. Symptomatic DPN was mainly associated with an increase in *ρ* [97], while manifest ATTRv amyloidosis was associated with an increase in both *ρ* and T2_app_ [53]. *ρ* in the sciatic nerve at thigh level was already increased in early stages of DPN, similar to the increase in presymptomatic var*TTR*-carriers. Furthermore, *ρ* was found to correlate with disease severity in DPN by predicting higher symptom severity with an odds ratio of 2.9 per 100 proton spins [97].

More recent MRN studies focused on the further differentiation between type 1 (T1D) and type 2 diabetes (T2D). While nerve lesions in T1D were predominantly T2w hyperintense, T2D was associated with an increase in T2w hypointense lesions [99]. As the evaluated MRN sequences were fat-saturated, T2w hypointense lesions presumably represent fat deposits within nerve tissue. An increase in both lesion types correlated with increasing disease severity. In addition, T2w hyperintense lesions in T1D positively correlated with serologic markers for hyperglycemia (i.e., HbA1c levels) and impaired NCS, while T2w hypointense lesions in T2D correlated with serologic markers for dyslipidemia [99]. These results were supported by studies showing that hyperglycemia-induced accumulation of advanced glycation end products in the extracellular matrix has been linked to the destruction of myelin and impaired axonal regeneration after damage [100]. Microvascular or intraneural lipid deposition might contribute to DPN development in T2D, potentially explaining why glycemic control alone often fails to improve DPN in T2D but not in T1D [101]. The influence of serum cholesterol levels on the occurrence of peripheral nerve lesions in patients with T2D as identified by MRN was further investigated and showed that decreased low-density lipoprotein (LDL) levels were associated with more T2w hypointense, lipid equivalent lesions to the sciatic nerve combined with an increased nerve CSA [102]. Patients with higher lesion numbers and lower LDL levels were found to have more advanced DPN symptoms and worse NCS. Even though some studies reported a beneficial effect on DPN progression when cholesterol levels are lowered [103, 104], other studies see detrimental effects on neuropathic symptoms and microvascular damage when serum cholesterol levels are decreased by statins and are, therewith, in line with the MRN correlation report further supporting the functional relevance of T2w hypointense lesions [105–107]. Proximal T2w hyperintense fascicular nerve lesions, on the other hand, were found to be associated with painful DPN related to both T1D and T2D, indicating that pain in DPN is not exclusively caused by changes in peripheral nociceptors and central sensitization but also by extensive structural damage to peripheral nerve fascicles [108].

Dorsal root ganglia (DRG), the most proximal postsynaptic component of the PNS, seemingly play an important role in DPN, but can only be analyzed postmortem. Animal studies found a significant correlation between a decrease in DRG volume and severity of neuropathic symptoms [109, 110]. In an MRN approach, human DRGs were visualized in vivo, showing significantly smaller DRG volumes in patients with severe DPN compared to patients with only mild or moderate symptoms [111]. A T2w signal increase in DRG was additionally found in DPN patients, but the relevance remains unclear as it did not correlate with DRG volumes. High triglyceride levels were associated with lower DRG volumes; however, a negative correlation between LDL cholesterol levels and DRG volumes contradicted previous study results and require further investigation [102, 111].

Besides the evaluation of T2w hypo- and hyperintense lesions, and the quantitative markers *ρ* and T2_app_, DTI is a method that has shown to be beneficial for nerve lesion detection in patients with DPN; especially the FA was identified as a highly sensitive parameter for structural nerve damage in T1D and T2D [5–112]. Sciatic nerve FA was recently proposed as a surrogate parameter for nerve integrity in DPN (Fig. 114) after it correlated well with electrophysiologic measurements of distal lower limb and hand nerves in symptomatic and presymptomatic diabetic patients [115]. The authors concluded that the FA is not only functionally relevant, but also that proximal nerve damage parallels distal nerve function even before patients start to experience clinical PNP symptoms. Combined with other quantitative MRN markers, *ρ* and FA might have the potential to predict disease progression in DPN and other neuropathies.

**Fig. 5:**
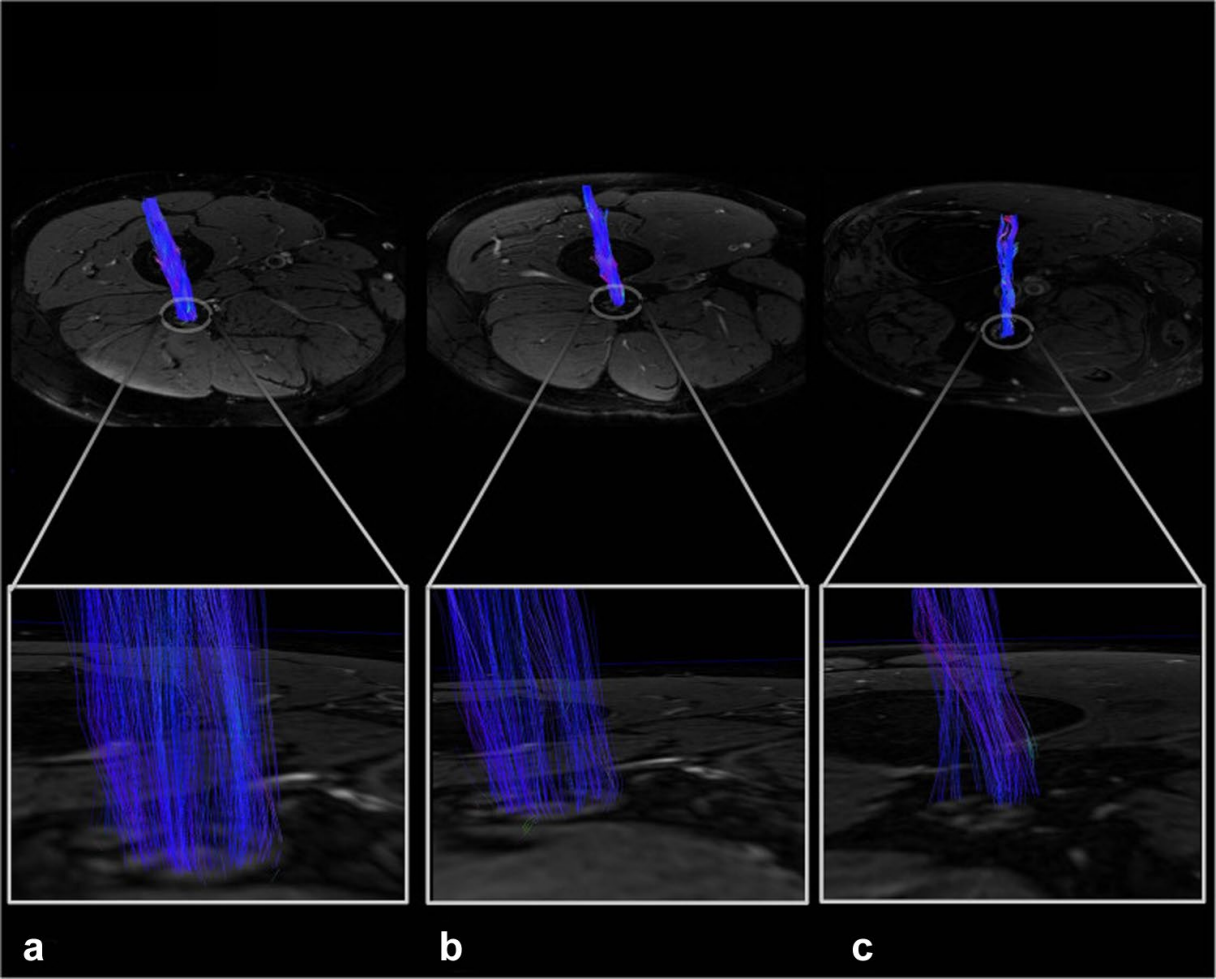
Representative sciatic nerve fiber tracts. Reconstructed, 3-dimensional fiber tracts of the sciatic nerve with color encoding according to the DTI eigenvector color map where voxel color reflects the direction of the diffusion tensor in that voxel and intensities are scaled with the FA. Nerve fiber tracts in the control are dense and contiguous (**A**), which decreases already in the patient with prediabetes (**B**), and even more in the patient with DPN (**C**). From Jende et al. [115] under the Creative Commons Attribution license

### MRN Results in Other Diffuse Neuropathies

Since the first groundbreaking applications of MRN in ATTRv amyloidosis and DPN, other diffuse neuropathies have been evaluated, and selected studies will be briefly reported here.

#### Systemic Light-Chain Amyloidosis

In a study conducted in untreated patients with systemic light-chain amyloidosis, MRN revealed similar results to the ones described in ATTRv amyloidosis [116]. Nerve lesions, as defined by an increased T2w signal and CSA, were predominantly found proximally within the sciatic nerve at thigh level. Signal quantification at the distal thigh confirmed *ρ* as the most reliable marker that can differentiate between different disease severities, as it was previously reported for DPN. T2_app_ was additionally increased in more advanced clinical cases only. This distinct alteration of quantitative MRN parameters in ATTRv and light-chain amyloidosis, especially the early and strong increase of *ρ*, supports the hypothesis that microstructural changes in the extracellular nerve matrix, potentially caused by amyloid deposits and not edema, are the driving force of the observed T2w signal increase [51]. As patients with light-chain amyloidosis might develop a PNP either due to the amyloid disease itself or as a side-effect from neurotoxic therapies, the future potential of MRN might lay in the identification of patients at risk for PNP development.

#### Chronic Inflammatory Demyelinating Polyneuropathy

In chronic inflammatory demyelinating polyneuropathy (CIDP), several MRI studies reported on a caliber increase of the brachial or lumbosacral plexus, often combined with an increased nerve T2w signal [117, 118]. However, results are inconsistent and the described changes at plexus level were only detectable in up to 86% of all patients. In a study applying MRN with large anatomical coverage of upper and lower extremity nerves, nerve CSA was again increased in a subset of patients only, but strong correlations between CSA values and electrophysiologic parameters of demyelination (i.e., F wave latencies and NCVs) were observed [119]. While not correlating well with NCS, nerve T2w signal was also found to be increased in CIDP. Both pathologic alterations were found predominantly in the proximal sections of upper and lower extremity nerves. Further signal quantification revealed that the observed T2w signal increase was mainly induced by an increase in *ρ* rather than in T2_app_, indicating an increase of macromolecular bound water molecules similar to findings described in DPN [97]. Even though nerve T2w signal and *ρ* were found to have high diagnostic accuracy, the authors concluded that CSA might be the most reliable parameter as it correlated with NCS, and might, therewith, complement other diagnostics in CIDP patients. DTI seemingly also contributes to a better diagnosis in CIDP; An observed decrease of the FA in CIDP patients was found to be the result of an increase of the RD, both known to be markers of myelin sheath integrity, while the AD, a marker of axonal integrity, did not differ between CIDP patients and healthy controls [120].

#### 5q-Linked Spinal Muscular Atrophy

A study conducted in adult patients with 5q-linked spinal muscular atrophy (SMA) found a marked decrease of nerve calibers as measured by CSA, which was most pronounced in the spinal nerves but was also observable for the lower extremity nerves, representing nerve atrophy [121]. While nerve T2w signal was only elevated at level of the spinal nerves, T2-relaxometry revealed a significant increase in T2_app_ and reduction in *ρ* for the tibial and peroneal fascicles within the distal sciatic nerve. The observed alteration of microstructural markers in SMA was opposed to the alterations in the previously analyzed length-dependent PNPs. While the increase of *ρ* in PNPs was hypothesized to be caused by protein deposits or inflammatory reactions in the extracellular matrix, the marked decrease of *ρ* in SMA in combination with the observed nerve atrophy well reflects the underlying pathomechanism in SMA with the decay of lower motor neurons and subsequent axonal loss. In amyotrophic lateral sclerosis (ALS), an acquired motor neuron disease, nerve CSA was not markedly reduced compared to controls [122]. However, the patients included in that study were at early disease stages, while SMA study participants had a long-standing disease with chronic structural transformations of their nerves. Quantitative markers were not evaluated in ALS, but the finding of an unaltered CSA in ALS was identified as an important criterion to distinguish ALS from multifocal motor neuropathy, which accounts for frequent diagnostic challenges in early disease stages. In SMA, sciatic nerve MTR was additionally evaluated and was found to correlate well with clinical symptom scores and CMAPs [123]. MTR was proposed as a novel imaging biomarker that might have the potential to measure the early stages of the regenerative process inside motor neurons in patients treated with one of the new pharmacotherapies. In combination with *ρ* and T2_app_, monitoring of structural nerve integrity in SMA patients under causal therapies might be possible in the future.

#### Charcot-Marie-Tooth Disease

Advanced MRN imaging techniques have also been utilized in patients with Charcot-Marie-Tooth disease (CMT) with promising results. DTI was identified as a useful tool to improve the detection of nerve abnormalities in patients with CMT type 1 [124]. Specifically, the FA was lower, and the apparent diffusion coefficient (ADC) was higher in CMT patients compared to healthy controls. *ρ* was also altered in CMT patients, but did not represent the severity of the neuropathy, and T2_app_ failed to show any differences between CMT and controls. DTI parameters, on the other hand, correlated well with clinical scores and NCS, indicating the functional relevance and making it an advantageous tool for quantifying nerve injury in CMT patients. Sciatic nerve MTR was proposed as another promising biomarker in patients with CMT type 1 and 2, as it is significantly decreased in these patients compared to controls and showed a good correlation with clinical disability scores [125]. The interscan and inter-rater reliability was also high, making it a valid and highly repeatable tool that can aid to improve the diagnosis and estimate the disease severity. This is particularly important in CMT, where distal nerves are often fully degenerated and proximal nerves are hardly amenable by conservative diagnostics.

## Achievements and Future Implications

The results from the presented studies on the relatively young method MRN have enhanced the understanding of peripheral neuropathies and also improved patient management. MRN allows the imaging of the PNS with high structural resolution down to the level of single nerve fascicles and provides large anatomic coverage at the same time. In focal neuropathies, MRN has proven that it can overcome typical limitations of conventional diagnostics by precisely determining the exact lesion localization, which has already impacted the therapeutic decision making, especially in unclear or atypical cases. Delayed diagnoses or unnecessary, often invasive, procedures can be prevented, and surgical treatments can be more focused, consequently, leading to better patient outcomes.

MRN has also led to a better understanding of diffuse neuropathies as the direct and non-invasive visualization of nerve tissue in vivo enabled the characterization of nerve lesion pattern in various neuropathies. While studies on mononeuropathies mainly relied on the interpretation of the nerve T2w signal, quantitative MRN markers, whether signal dependent or morphometric, seem to be advantageous when evaluating diffuse neuropathies. Even though an intraneural T2w signal increase is highly sensitive, it is unspecific and does not allow the differentiation between different etiologies. Determining the etiology of a neuropathy is still not possible by MRN alone and relies on clinical input, but recent results show some promising specificity. Particularly the quantitative microstructural markers, *ρ* and T2_app_, might contribute to a better differentiation of disease entities by reflecting the underlying pathomorphology. In polyneuropathies, *ρ* shows a strong and early increase even at presymptomatic disease stages, while T2_app_ shows an additional increase only in more advanced disease stages [53, 85, 97, 116]. It is hypothesized that the *ρ* increase is mainly caused by a change in the macromolecular organization of the extracellular matrix as it can be caused for example, by an accumulation of amyloid deposits in hereditary or systemic amyloidosis, advanced glycosylated end products in diabetes, or cytotoxic proteins in alcohol-related PNP. Nerve lesions in SMA, a motor neuron disease, were characterized by a decrease in *ρ* (opposing to the results in PNPs), and an increase in T2_app_ (similar to the results in PNPs), which can be well explained with the decay of lower motor neurons and subsequent axonal loss in SMA [52]. On the contrary, a MRN study conducted in MS patients (not subject of this review) that detected an involvement of the PNS in this demyelinating disease, reported an increased *ρ* (similar to the results found in PNPs) and a decreased T2_app_ (opposing to the results in PNPs) in lower extremity nerves [53]. An inflammatory process leading to an impaired blood-nerve barrier with subsequent leakage of plasma proteins was favored as a potential explanation. In addition, a post-mortem study found an increased *ρ* in demyelinated areas in the CNS of MS patients [97], so that a potential peripheral co-demyelination was hypothesized as the origin of the observed increase in sciatic nerve *ρ* [108]. However, the different diseases that directly or indirectly affect the PNS are easily distinguishable by their clinical presentation, and diagnosis does not rely on MRN, e.g., SMA will not be misdiagnosed as a PNP. However, within different neuropathy groups, differentiation can be more challenging, and MRN might be able to assist in establishing the correct diagnosis. As examples, nerve lesion in ATTRv amyloidosis appear to be more diffuse [120], while the spatial distribution in DPN is multifocal and clustered [121], CSA and T2w signal increase at the level of the plexus, and the spinal nerves are more pronounced in CIDP [126] than in ATTRv amyloidosis or DPN, and nerve lesions in T1D are predominantly T2w hyperintense, while they appear to be more T2w hypointense in T2D [126].

Even though these results are very promising, they are still based on group comparisons. While therapeutic decisions can be made based on MRN results in individual patients with mononeuropathies, it remains difficult when it comes to diffuse neuropathies. The biggest challenge going forward will be the transfer of MRN results from group comparisons down to an individual patient level. Research should focus on quantitative MRN markers that have the potential to become sensitive new imaging biomarkers for both an earlier detection of nerve injury and for the monitoring of structural nerve damage under disease modifying therapies, which can be well explained by taking ATTRv amyloidosis as an example. Detecting nerve injury at an early, even presymptomatic stage would be beneficial for still asymptomatic var*TTR*-carriers that often loose valuable time before a causative therapy is commenced, simply because objective signs of manifest ATTRv amyloidosis can be difficult to capture clinically or by NCS. Here, MRN might identify var*TTR*-carriers that are likely to develop the symptomatic disease in the foreseeable future, either by determining a certain threshold of nerve lesions that needs to be exceeded before first symptoms occur or by detecting a certain change in quantitative markers in regular follow-up examinations (e.g., a constant rise in *ρ* or decrease in MTR). In more advanced stages of ATTRv amyloidosis, it is often difficult to determine whether a patient benefits from the chosen therapy or not, because electrophysiologic potentials are no longer elicitable. However, such information would be particularly interesting and directly influence the further therapeutic approach as several different causative therapies are now available [82–84]. New imaging biomarkers might help to detect progressive structural nerve damage under therapy sooner and could drive adjustments in treatment, consequently, leading to better outcomes. Preliminary data in a small cohort were presented in a conference paper [127]. Measuring the *ρ* over several years by performing follow-up MRN, the *ρ* reportedly increased significantly in previously asymptomatic var*TTR*-carriers who developed clinically symptomatic ATTRv amyloidosis during the conduct of the study, while var*TTR*-carriers who remained asymptomatic presented with stable *ρ* values. In patients with symptomatic ATTRv amyloidosis clinical stability or disease progression also correlated with the sciatic nerve *ρ* [127]. However, longitudinal studies in larger cohorts are needed to verify, whether *ρ* is a valid biomarker to detect disease progression or improvement, and potential confounding factors need to be considered. Future studies should also focus on diseases other than ATTRv amyloidosis, and include other quantitative MRN markers such as MTR, FA, or CSA, as a combination of different imaging biomarkers might lead to the most robust results.

## Supplementary Information

Below is the link to the electronic supplementary material.

Supplementary file1 (PDF 499 KB)
